# An improved hybrid artificial bee colony algorithm for a multi-supplier closed-loop location inventory problem with customer returns

**DOI:** 10.1371/journal.pone.0324343

**Published:** 2025-05-22

**Authors:** Hao Guo, Xiaomei Lai, Ju Guo, Ge You, Ibrahim Alnafrah

**Affiliations:** 1 School of Management, Wuhan Textile University, Wuhan, Hubei, China; 2 Research Center of Enterprise Decision Support, Key Research Institute of Humanities and Social Sciences in Universities of Hubei Province, Wuhan, Hubei, China; 3 College of International Business and Economics, Wuhan Textile University, Wuhan, Hubei, China; 4 Hubei Key Laboratory of Digital Textile Equipment, Wuhan Textile University, Wuhan, Hubei, China; 5 School of Literature and Media, Nanfang College Guangzhou, Guangzhou, Guangdong, China; 6 Graduate School of Economics and Management, Ural Federal University, Yekaterinburg, Russia; 7 Institute of Graduate Studies and Research, Cyprus International University, Nicosia, North Cyprus; Wenzhou University College of Mechanical and Electrical Engineering, CHINA

## Abstract

Customer returns are an unavoidable and increasingly costly challenge in business operations, especially in online marketplaces. This study addresses this issue by introducing a practical multi-supplier closed-loop location-inventory problem (CLLIP) that incorporates customer returns. The objective of the CLLIP is to minimize overall supply chain costs by optimizing facility location and inventory management strategies. To solve this complex problem, an improved hybrid artificial bee colony algorithm (IHABC) is proposed, which integrates two novel search equations to generate candidate solutions during the employed bee and onlooker bee phases, effectively balancing exploration and exploitation. The performance of IHABC is evaluated against various artificial bee colony variants as well as the commercial solver Lingo. The results of numerical experiments demonstrate that IHABC consistently outperforms competing methods, achieving superior solutions with the lowest mean values and optimal total cost results, while also requiring less computation time. The results of numerical experiments demonstrate that IHABC consistently outperforms competing methods, achieving up to 29.97% improvement in solution quality over the standard ABC algorithm. These findings confirm that IHABC is a highly effective and efficient tool for solving the proposed CLLIP. Furthermore, a sensitivity analysis is conducted to provide actionable insights, enabling managers to make informed and strategic decisions in real-world supply chain operations.

## 1. Introduction

The rapid growth of online sales has driven a surge in customer returns, particularly false failure returns [1]. This trend not only results in substantial financial losses for businesses but also leads to significant resource wastage on a societal level [2]. According to the National Retail Federation’s 2023 report, U.S. retailers lost over $743 billion due to product returns, with the average retailer facing $145 million in returns for every $1 billion in sales [3]. Online sales return rates are significantly higher, ranging from 20% to 30%, which is two to three times higher than those of brick-and-mortar stores [4]. For instance, JD, a leading B2C online retailer in China, experiences a return rate exceeding 10%, with annual return values reaching hundreds of billions of dollars [5]. To handle this growing volume of returns, businesses must implement efficient closed-loop supply chain (CLSC) systems. Moreover, regular adjustments and continuous optimization of supply chain networks are essential to maintaining profitability and operational efficiency while addressing the sustainability challenges posed by increased product returns.

The design of a CLSC network is a critical determinant of cost reduction and customer service level improvement [6]. Empirical evidence suggests that a well-structured network can lower supply chain costs by up to 60% [7]. In a dynamic marketplace, companies are compelled to continuously optimize both strategic and tactical supply chain decisions to maintain competitiveness. Strategic decisions, such as facility location, focus on maximizing efficiency, while tactical decisions, including inventory control, aim to improve responsiveness [8]. Integrating joint location-inventory models is essential for avoiding suboptimal designs that could significantly compromise logistics system performance[8,9]. Therefore, a thorough examination of closed-loop location-inventory problems is indispensable for making informed decisions regarding customer returns and overall network efficiency.

Concurrently, relying on a single supplier poses significant risks, such as high dependency, limited flexibility, and an inability to manage unexpected demand surges, often resulting in supply disruptions [10]. The COVID-19 pandemic highlighted these vulnerabilities, as many suppliers were forced to cease operations, triggering crises for businesses reliant on single-source suppliers [11]. To mitigate these risks, adopting a multi-supplier strategy is essential [12]. In this study, the enterprise collaborates with multiple suppliers, a practice that not only reduces the risk of supply disruptions but also enhances product quality and stabilizes pricing. Thus, investigating multi-supplier CLLIP becomes vital for guiding both strategic and tactical decisions regarding customer returns, improving supply chain resilience, and strengthening the enterprise’s competitive advantage.

The motivation for this research stems from the pressing challenges posed by the rising volume of customer returns in modern supply chains, particularly in e-commerce. With return rates significantly higher in online retail compared to traditional stores, businesses face substantial financial losses and operational inefficiencies. Existing supply chain models often overlook the integration of customer returns into optimization frameworks, leading to suboptimal decision-making in facility location, inventory management, and supplier selection. Moreover, reliance on single suppliers exposes firms to heightened risks of disruptions, as seen during the COVID-19 pandemic. To address these gaps, this study introduces a novel multi-supplier closed-loop location-inventory problem (CLLIP) that explicitly incorporates customer returns, offering a more resilient and cost-effective approach to supply chain design. By developing an improved hybrid artificial bee colony (IHABC) algorithm, this research provides an advanced optimization technique capable of efficiently solving the complex CLLIP. The findings contribute to both theory and practice, offering actionable insights for businesses seeking to enhance profitability, sustainability, and resilience in an increasingly dynamic market.

In this study, we propose an integrated approach to incorporate customer returns into the multi-supplier location-inventory problem within a CLSC. This approach aims to help firms better cope with the increasing volume of returns, optimize their supply chain networks, improve operational efficiency and reduce costs, and thus maintain an edge in a competitive marketplace. The CLSC framework comprises potential suppliers, candidate hybrid distribution-collection centers (HDCCs), and designated customer zones. The proposed CLLIP is formulated as a mixed-integer nonlinear programming model, aiming to minimize the total cost of the supply chain system. This model addresses several critical decisions, including the number and selection of suppliers, the location and quantity of candidate HDCCs, the transportation flow between suppliers and HDCCs, the assignment of customer zones to facilities in both forward and reverse logistics, and the size and timing of orders for each HDCC. Given the inherent complexity of this problem, we develop an improved hybrid artificial bee colony algorithm (IHABC), which integrates the artificial bee colony algorithm (ABC) with the differential evolution algorithm (DE). This hybrid approach is designed to efficiently achieve near-optimal solutions within a computationally feasible timeframe.

This paper aims to investigate the following research questions: (1) How can an effective optimization model be formulated for joint decisions in the CLLIP while considering customer returns and multiple suppliers? (2) What are the optimal number and locations of facilities, supplier selections, customer allocations, and inventory policies within a closed-loop system that accounts for customer returns? (3) How do cost factors influence the overall performance of the CLSC under study? To address these questions, we propose a mixed-integer nonlinear programming model, which is subsequently solved using the IHABC. Additionally, the accuracy and efficiency of IHABC are evaluated through a series of numerical experiments, followed by a comprehensive analysis and discussion of the results.

This study makes significant contributions from methodological, managerial, and theoretical perspectives: (i) Theoretical Contribution: A novel multi-supplier closed-loop location-inventory model is developed to address CLLIP by incorporating customer returns and multiple suppliers, extending the traditional frameworks. (ii) Methodological Contribution: IHABC introduces innovative improvement strategies in both the employed bee and onlooker bee phases, enhancing the efficiency of the artificial bee colony (ABC) algorithm. Various algorithms, including ABC, GABC, CABC, qABC, and Lingo, are utilized to tackle the complex CLLIP, and numerical experiments demonstrate that IHABC outperforms other intelligent algorithms in terms of both effectiveness and computational efficiency when applied to the proposed model. (iii) Managerial Contribution: The performance of IHABC is analyzed under different key parameters, providing valuable insights for optimizing facility locations and inventory strategies, thereby enhancing cost-effectiveness and sustainability in supply chain management.

The remainder of the study is organized as follows: Section 2 offers a systematic literature review. Section 3 presents the research problem and formulates the CLLIP model. Section 4 introduces an efficient IHABC for solving the model. Section 5 presents the numerical study and computational results. Finally, Section 6 concludes and outlines further research directions.

## 2. Literature review

Location-inventory problems (LIPs) are recognized for their NP-hard complexity and have received considerable attention in academic literature. Daskin et al. [13] proposed a seminal model that integrates operating inventory, safety stock, and economies of scale into facility location decisions, thereby establishing a foundational class of location-inventory models. For instance, the inclusion of economies of scale in facility location decisions enables firms to achieve cost savings through centralized operations, which can be passed on to consumers in the form of lower prices. Similarly, the consideration of safety stock helps firms mitigate the risks associated with demand variability, thereby enhancing supply chain resilience and reducing the likelihood of stockouts. These benefits are particularly relevant in industries with high demand uncertainty, such as retail and manufacturing, where the ability to balance cost and service levels is a key determinant of competitive advantage. Building on this foundational work, numerous researchers have further examined LIPs and developed efficient algorithms to solve them.

Recent studies on LIPs have embraced diverse perspectives, reflecting the growing complexity and relevance of this field Comprehensive literature reviews by Farahani et al. [14] and Jalal et al. [15] have synthesized existing research in this area, providing the foundation for further investigations Furthermore, contemporary studies have expanded the scope of LIPs, adapting them to address increasingly complex real-world challenges. For instance, Saha et al. [16] investigated a location-inventory model that incorporates the risks of partial disruptions caused by consumer preferences for substitute products and the occurrence of backorders, offering a more nuanced understanding of demand variability and supply chain resilience In a separate study, Araya et al. [17] developed multi-commodity location-inventory models that combine both continuous and periodic review inventory control strategies, alongside modular stochastic capacity constraints, thereby addressing the challenges of multi-product supply chains with fluctuating demand Additionally, Fathi et al. [18] developed an integrated optimization model for location-inventory problems by classifying customer orders into priority and regular categories, highlighting the importance of differentiated service levels in supply chain management. Wang et al. [19] introduced a location-inventory delivery model that incorporates a joint replenishment policy, optimizing the coordination between inventory management and transportation logistics. Camacho-Vallejo & Dávila [20] examined a bi-level warehouse location problem that integrates inventory decisions, developing an effective nested evolutionary algorithm to tackle it. Mohammadi et al. [21] studied a location-inventory model for a four-level sustainable supply chain of a perishable product with price-dependent demand and deterioration rates. Tapia-Ubeda et al. [22] proposed a novel location-inventory problem that emphasizes supplier selection decisions as a critical factor, underscoring the strategic importance of supplier relationships in supply chain design. Additionally, Li et al. [23] explored the location-inventory problem, concentrating on the implications of economies and diseconomies of scale, providing insights into the cost dynamics of scaling supply chain operations.

Recently, LIPs have been expanded to incorporate closed-loop supply chains, reflecting the growing emphasis on sustainability and resource efficiency in supply chain management. Diabat et al. [24] proposed a closed-loop location inventory model in which returned products are remanufactured into spare parts and distributed back to retailers, highlighting the potential of value recovery in reverse logistics. Similarly, Zhang & Unnikrishnan [9] addressed a coordinated inventory-location problem in a closed-loop supply chain, focusing on the challenges posed by uncertain demand. Li et al. [25] investigated the joint optimization of location and inventory decisions within a closed-loop system that incorporates third-party logistics, emphasizing the role of external partners in enhancing supply chain efficiency In a subsequent study, Li et al. [26] focused on a multi-commodity location-inventory problem, specifically addressing false failure returns within a forward-reverse logistics network, thereby addressing the complexities of product returns in multi-product environments Guo et al. [27] examined a location-inventory problem in a closed-loop supply chain, considering both new and used product sales across primary and secondary markets. Furthermore, Becerra et al. [28] optimized the location, inventory, and transportation decisions in a sustainable closed-loop supply chain, integrating environmental considerations into supply chain design. Similarly, Guo et al. [29] examined a multi-commodity closed-loop location-inventory problem, focusing on commercial product returns and their impact on supply chain performance. Despite these significant advancements, research on multi-supplier closed-loop location-inventory problems with customer returns remains limited, representing a critical gap in the literature that this study seeks to address.

Several effective methodologies have been proposed to address the challenges associated with LIPs. Given their inherent NP-hard complexity, a variety of heuristic algorithms have been developed, including Genetic Algorithm (GA) [18], □-optimal Lipschitz optimization algorithm [19], nested evolutionary algorithm [20], differential evolution algorithm (DE) [25,29], hybrid harmony search [30]. Additionally, the artificial bee colony algorithm (ABC) [31] has gained significant attention for its robust performance in addressing NP-hard optimization problems. The performance of ABC has been thoroughly investigated across various domains, including the distributed resource-constrained hybrid flow shop problem [32], flexible job shop scheduling problem [33], network communication security [34], flowshop scheduling problem [35], and replenishment-distribution problem [36]. However, its potential for solving mixed-integer nonlinear programming (MINLP) problems remains underexplored. To bridge this gap and enhance its capabilities, it is essential to develop an efficient algorithm specifically tailored for the novel multi-supplier CLLIP presented in this study.

Despite the extensive research on LIPs, most existing studies focus on either single-supplier systems or ignore the role of customer returns in CLSC environments. While numerous heuristic and metaheuristic algorithms have been employed to solve LIPs, their effectiveness in handling multi-supplier MINLP problems remains largely unexplored. Specifically, the artificial bee colony (ABC) algorithm has demonstrated strong optimization capabilities in various domains but has not been systematically adapted to solve the complex multi-supplier CLLIP with customer returns. This study addresses these gaps by integrating multi-supplier considerations into the CLLIP framework and enhancing the ABC algorithm with novel search strategies. Table 1 provides a comparative overview of related studies, highlighting the unique contributions of our model.

**Table 1:** Summary of related works.

Author(s)	Model	Application	Supplier		Inventory	Algorithm
			Single	Multiple		
Saha et al. [16]	LIP	General		✓	✓	Modified particle swarm optimization
Fathi et al. [18]	LIP	General	✓		✓	Hybrid genetic algorithm
Wang et al. [19]	LIP	General		✓	✓	Iterative heuristic
Camacho-Vallejo & Dávila [20]	LIP	General			✓	Nested evolutionary algorithms
Li et al. [25]	CLLIP	General	✓		✓	Improved hybrid differential evolution
Li et al. [26]	CLLIP	General	✓		✓	Improved differential evolution
Guo et al. [29]	CLLIP	General	✓		✓	Hybrid adaptive differential evolution
Dai et al. [30]	LIP	Perishable products		✓	✓	Hybrid genetic algorithm
Guo et al. [27]	CLLIP	General	✓		✓	Improved self-adaptive differential evolution
Guo et al. [37]	CLLIP	General	✓		✓	Modified hybrid differential evolution
This paper	CLLIP	General		✓	✓	Improved hybrid artificial bee colony

## 3. Problem formulation

### 3.1. Problem description

This paper explores a multi-period, multi-stage supply chain network consisting of multiple suppliers, candidate HDCCs, and customer zones. In this integrated system, suppliers function as both production and recovery centers, whereas HDCCs operate as hybrid facilities that combine distribution and collection functions. As aforementioned, establishing suppliers and HDCCs offers advantages such as significant cost reductions and minimized environmental impact, primarily due to the shared use of material handling equipment and infrastructure [37]. This integrated approach not only enhances operational efficiency but also supports sustainability by consolidating forward and reverse logistics activities into a unified framework. Furthermore, the multi-period nature of the model allows for dynamic decision-making, enabling firms to adapt to changing market conditions and demand patterns over time.

**Fig 1:**
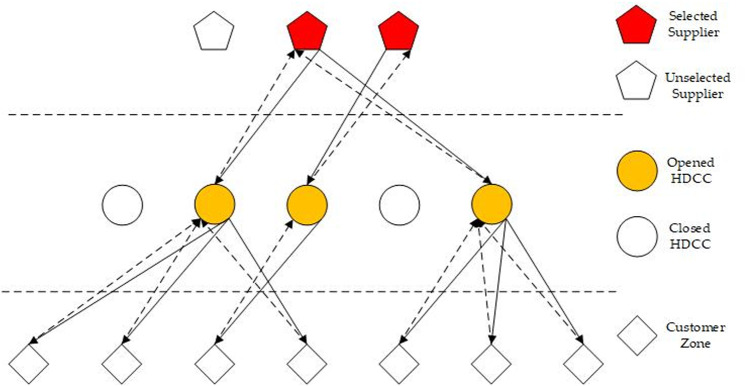
The closed-loop supply chain of the proposed problem.

The structure of the CLSC network is depicted in Fig 1. In the forward flow, selected suppliers deliver products to a set of customer zones via HDCCs to meet the demand of each customer zone. In the reverse flow, HDCCs collect returns from customer zones. After inspection, returns are categorized into true failure returns and false failure returns [29]. False failure returns are reconditioned by the HDCCs and then re-enter the forward flow, effectively reducing waste and maximizing resource utilization. True defective returns are sent back to suppliers through HDCCs for repair, recovery, or remanufacturing, ensuring that defective products are properly handled and reintegrated into the supply chain. It is noteworthy that customer returns, after undergoing the necessary processing, are forwarded to customer zones as new products [38], thereby closing the loop and maintaining product availability.

Specifically, Fig 1 involves 7 customer zones, 5 candidate HDCCs, and 3 candidate suppliers. Candidate Suppliers 2 and 3 are selected, and candidate HDCCs 2, 3, and 5 are opened. In the first layer, candidate HDCCs 2 and 5 are assigned to supplier 2, while candidate HDCC 3 is assigned to supplier 3. In the second layer, customer zones 1, 2, and 4 are allocated to HDCC 2; customer zone 3 is allocated to HDCC 3; and customer zones 5, 6, and 7 are allocated to HDCC 5.

This paper presents a novel multi-supplier CLLIP in the presence of customer returns. This issue integrates facility location and inventory control decisions within a CLSC, formulated through a mixed-integer nonlinear programming (MINLP) model. The model’s objective is to select suppliers, determine the optimal locations for suppliers and HDCCs, assign customer zones to HDCCs, and link HDCCs to suppliers. Furthermore, the model aims to optimize the inventory policies implemented by HDCCs, with the overarching goal of minimizing total costs in such a CLSC network. By simultaneously addressing strategic decisions (e.g., facility location and supplier selection) and operational decisions (e.g., inventory control and customer allocation), this model provides a comprehensive framework for managing the complexities of multi-supplier CLSCs. The inclusion of customer returns adds a layer of realism, ensuring that the model captures the challenges and opportunities associated with reverse logistics.

The model is formulated based on the key mechanisms of the real-world practice under study. For simplicity, the following assumptions are made.

- Shortages are not permitted.
- Each customer zone is served by only one HDCC.
- The replenishment lead time is constant.
- Only a single item is supplied.
- Customer zones are predetermined and fixed.
- The return rate is predetermined.
- Returns from each customer zone are collected by one HDCC.

Assumptions 1–4 are commonly adopted by most multi-echelon LIP models, as they simplify the model for easier solution. Assumption 5 is based on the perspective of location decisions, assuming that customer zones are predetermined and fixed, which ensures a stable demand structure for planning purposes. Assumptions 6 and 7 enable efficient planning of reverse logistics activities.

#### 3.1.1 Notation.

Sets:

- *S = *set of candidate locations for suppliers;
- *R* = set of candidate locations for HDCCs;
- *I* = set of customer zones.

Parameters:

- *a*_*r*_ = fixed (annual) cost of building and operating a HDCC at location *r*, for each *r* ∈ *R*;
- *a*_*s*_ = fixed (annual) cost of selecting a supplier at location *s*, for each *s* ∈ *S*;
- *b*_*sr*_ = fixed administrative and handling cost of placing an order at HDCC *r* to the supplier *s*, for each *s* ∈ *S*, *r* ∈ *R*;
- *c*_*sr*_ = fixed shipment cost of a shipment between the supplier *s* and HDCC *r*, for each *s* ∈ *S*, *r* ∈ *R*;
- *d*_*ri*_ = shipment cost per unit between HDCC *r* and customer zone *i*, for each *r* ∈ *R*, *i* ∈ *I*;
- *e*_*sr*_ = unit variable shipment cost between the supplier *s* and HDCC *r*, for each *s* ∈ *S*, *r* ∈ *R*;
- *f*_*r*_ = processing cost per unit of customer returns at HDCC *r*, for each *r* ∈ *R*;
- *h*_*r*_ = (annual) holding cost per unit at HDCC *r*, for each *r* ∈ *R*;
- *q*_*i*_ = return rate at customer zone *i*, for each *i* ∈ *I*;
- *µ*_*i*_ = mean (daily) demand at customer zone *i*, for each *i* ∈ *I*;
- $\sigma_{i}^{2}$ = variance of (daily) demand at customer zone *i*, for each *i* ∈ *I*;
- *α* = desired percentage of fulfilled market demand (fill rate);
- *z*_*α*_ = standard normal deviate such that *P* (*z* ≤ *z*_*α*_) =*α*;
- *L* = order lead time (in days) at HDCCs;
- *λ* = working days per year;
- *θ* = percentage of false failure returns;
- *β* = percentage of true failure returns, where *β*=1-*θ*.

Decision variables:

- *W*_*s*_ = 1 if a supplier at location *s* is selected, and 0 otherwise, for each *s* ∈ *S*;
- *X*_*r*_ = 1 if opening one HDCC at location *r*, and 0 otherwise, for each *r* ∈ *R*;
- *Y*_*sr*_ = 1 if supplier *s* is assigned to HDCC *r*, and 0 otherwise, for each *s* ∈ *S*, *r* ∈ *R*;
- *Z*_*ri*_ = 1 if HDCC *r* is assigned to customer zone *i*, and 0 otherwise, for each *r* ∈ *R*, *i* ∈ *I*.

### 3.2. Model formulation

The total costs in the CLSC under study consist of three main components: (1) location costs which include the fixed cost of selecting suppliers and locating HDCCs, as well as the delivery costs between HDCCs and customer zones; (2) inventory costs which include working inventory costs and safety stock costs. Working inventory costs comprise the fixed costs of placing orders at HDCCs, the shipping costs between the suppliers and HDCCs, and the holding cost; (3) processing costs, which encompass the expenses associated with inspecting, reconditioning, and recovering returned products.

#### 3.2.1. Location and delivery cost.

The main influences for making facility location decisions are the fixed location cost and the delivery cost between HDCCs and customer zones which usually be measured by distance [25,29]. Therefore, the location cost (*C*_*L*_) can be expressed as follows:


$$C_{L}=\sum_{s\in S}a_{s}W_{s}\text{+}\sum_{r\in R}a_{r}X_{r}\text{+}\lambda \sum_{r\in R}\sum_{i\in I}\left(1+q_{i}\right)\mu_{i}d_{ri}Z_{ri}$$

This formulation highlights the trade-off between the upfront investment in establishing facilities and the ongoing transportation expenses, which are critical considerations in designing an efficient and cost-effective supply chain network

#### 3.2.2. Inventory cost.

1. **Working inventory cost:** In this study, we assume that HDCCs use a (*Q*, *r*) inventory model with type I service [39] to represent the inventory control problem. This choice is common as it allows for the approximation of the (*Q*, *r*) model with the order quantity determined by an economic order quantity (EOQ) model [40]. Therefore, we employ an EOQ model to optimize the inventory decisions in this study. The total working inventory costs of HDCCs include (a) order cost; (b) shipping cost between the suppliers and the HDCCs; and (c) holding cost of the working inventory. Let *D*_*sr*_ be the annual demand from supplier *s* to HDCC *r* per year, then $\frac{D_{sr}}{Q_{sr}}$ will be the number of orders from supplier *s* to HDCC *r* per year. This formulation ensures that inventory decisions are optimized to balance ordering and holding costs, while also accounting for the transportation expenses associated with supplier-HDCC interactions.
  (1) ** Order cost.** Since the HDCC *r* processes the same number of orders, the fixed ordering cost can be shared among items within the same order. Therefore, the annual fixed cost of placing orders from supplier *s* to HDCC *r* is $b_{sr}\frac{D_{sr}}{Q_{sr}}$.
  (2) ** Shipping costs between the suppliers and HDCCs.** Given that false failure returns in new or good conditions are reused by HDCCs to fulfill customer orders, the shipping quantity from the suppliers to HDCCs is the difference between customer demands and false failure returns. Meanwhile, true failure returns are shipped from HDCCs to the suppliers for repair or recovery, the transportation cost of true failure returns from HDCC *r* to the supplier *s* should be added to the shipping cost. Hence, the annual shipping cost from supplier *s* to HDCC *r* is $c_{sr}\frac{D_{sr}}{Q_{sr}}+\lambda e_{sr}\sum_{i\in I}\left(1-\left(\theta -\beta \right)q_{i}\right)\mu_{i}Z_{ri}Y_{sr}$.

This approach ensures that both forward and reverse logistics costs are accounted for, providing a more accurate representation of the total shipping expenses in the CLSC network.

(3) **Holding cost of the working inventory.** The annual holding cost at HDCC *r* includes the holding costs of the products in both forward and reverse flows. Therefore, the holding cost in the closed-loop supply chain from the supplier *s* to HDCC *r* is $\frac{\lambda h_{r}\sum_{i\in I}\left(\left(1\text{+}q_{i}\right)\mu_{i}Z_{ri}Y_{sr}\right)Q_{sr}}{2D_{sr}}$.

Therefore, the total annual working inventory cost from the supplier *s* to HDCC *r* in a closed-loop supply chain is:


$$b_{sr}\frac{D_{sr}}{Q_{sr}}+c_{sr}\frac{D_{sr}}{Q_{sr}}+\lambda e_{sr}\sum_{i\in I}\left(1-\left(\theta -\beta \right)q_{i}\right)\mu_{i}Z_{ri}Y_{sr}+\frac{\lambda h_{r}\sum_{i\in I}\left(\left(1\text{+}q_{i}\right)\mu_{i}Z_{ri}Y_{sr}\right)Q_{sr}}{2D_{sr}}$$

It is straightforward to show that the optimal value of *Q*_*sr*_ that minimizes this expression is equal to $Q_{sr}^{\text{*}}\text{=}\sqrt{\frac{2\left(b_{sr}+c_{sr}\right)}{\lambda h_{r}\sum_{i\in I}\left(\left(1\text{+}q_{i}\right)\mu_{i}Z_{ri}Y_{sr}\right)}}D_{sr}$. The corresponding annual working inventory cost from the supplier *s* to HDCC *r* can be expressed as:


$$\mathrm{\backslash [}\sqrt{2\lambda h_{r}\left(b_{sr}+c_{sr}\right)\sum_{i\in I}\left(\left(1\text{+}q_{i}\right)\mu_{i}Z_{ri}Y_{sr}\right)}+\lambda e_{sr}\sum_{i\in I}\left(1-\left(\theta -\beta \right)q_{i}\right)\mu_{i}Z_{ri}Y_{sr}\mathrm{\backslash ]}$$

The total annual working inventory cost in the closed-loop supply chain can be expressed as follows:


$$\mathrm{\backslash [}C_{WI}\text{=}\sum_{s\in S}\sum_{r\in R}\sqrt{2\lambda h_{r}\left(b_{sr}+c_{sr}\right)\sum_{i\in I}\left(\left(1\text{+}q_{i}\right)\mu_{i}Z_{ri}Y_{sr}\right)}+\lambda \sum_{s\in S}\sum_{r\in R}\sum_{i\in I}e_{sr}\left(1-\left(\theta -\beta \right)q_{i}\right)\mu_{i}Z_{ri}Y_{sr}\mathrm{\backslash ]}$$

2. **Safety stock cost:** Using Eppen’s risk pooling effects [40], the safety stock required to ensure that stockouts occur with a probability of *α* or less is $z_{\alpha }\sqrt{L\sum_{i\in I}\sigma_{i}^{2}Z_{ri}}$. Therefore, the holding cost for the safety stock cost at HDCCs is $C_{SS}=\sum_{r\in R}h_{r}z_{\alpha }\sqrt{L\sum_{i\in I}\sigma_{i}^{2}Z_{ri}}$.

This formulation ensures that inventory decisions are optimized to minimize costs while accounting for both forward and reverse logistics activities, providing a comprehensive framework for managing inventory in a closed-loop supply chain.

#### 3.2.3. Processing cost.

The customer returns collected at HDCCs undergo processing activities such as inspecting, repackaging, sorting, and cleaning. The processing cost at HDCCs is $C_{P}=\lambda \sum_{r\in R}\sum_{i\in I}f_{r}q_{i}\mu_{i}Z_{ri}$.

Consequently, the MINLP model is employed to solve the following:


$$\begin{array}{c} \begin{array}{cc} & \;\min C_{TOT}=C_{L}+C_{WI}+C_{SS}\text{+}C_{P}\\ & =\sum_{s\in S}a_{s}W_{s}\text{+}\sum_{r\in R}a_{r}X_{r}\text{+}\lambda \sum_{r\in R}\sum_{i\in I}\left(1+q_{i}\right)\mu_{i}d_{ri}Z_{ri}\\ & \text{+}\sum_{s\in S}\sum_{r\in R}\sqrt{2\lambda h_{r}\left(b_{sr}+c_{sr}\right)\sum_{i\in I}\left(\left(1\text{+}q_{i}\right)\mu_{i}Z_{ri}Y_{sr}\right)}\\ & +\lambda \sum_{s\in S}\sum_{r\in R}\sum_{i\in I}e_{sr}\left(1-\left(\theta -\beta \right)q_{i}\right)\mu_{i}Z_{ri}Y_{sr}\\ & \text{+}\sum_{r\in R}h_{r}z_{\alpha }\sqrt{L\sum_{i\in I}\sigma_{i}^{2}Z_{ri}}\text{+}\lambda \sum_{r\in R}\sum_{i\in I}f_{r}q_{i}\mu_{i}Z_{ri} \end{array} \end{array}$$ (1)

Subject to:


$$\sum_{s\in S}W_{s}\geq 1;$$ (2)

$$\sum_{r\in R}X_{r}\geq 1;$$ (3)

$$\sum_{r\in R}Z_{ri}=1,\forall i\in I;$$ (4)

$$Y_{sr}\leq W_{s},\forall s\in S,\forall r\in R;$$ (5)

$$Z_{ri}\leq X_{r},\forall i\in I,\forall r\in R;$$ (6)

$$\sum_{r\in R}Y_{sr}\geq W_{s},\forall s\in S;$$ (7)

$$\sum_{i\in I}Z_{ri}\geq X_{r},\forall r\in R;$$ (8)

$$Z_{ri}\leq \sum_{s\in S}Y_{sr}\leq 1,\forall r\in R,\forall i\in I\text{;}$$ (9)

$$W_{s}=\left\{0,1\right\},\forall s\in S;$$ (10)

$$X_{r}=\left\{0,1\right\},\forall r\in R;$$ (11)

$$Y_{sr}=\left\{0,1\right\},\forall s\in S,\forall r\in R;$$ (12)

$$Z_{ri}=\left\{0,1\right\},\forall r\in R,\forall i\in I;$$ (13)

The objective function in Equation (1), aims to minimize the total annual cost in the CLSC under study. Equations (2) and (3) dictate that at least one supplier and HDCC must be built, respectively. Equation (4) means that each customer zone is precisely assigned to one HDCC. Equation (5) ensures that each HDCC must be assigned to an existing supplier. Equation (6) means that no customer zone can be allocated to a HDCC unless it is opened. Equations (7) and (8) signify that the supplier and HDCC will be operational once built. Equation (9) ensures that each customer zone will be served by only one HDCC and supplier. Equations (10) – (13) mean that decision variables are binary. This MINLP model provides a comprehensive framework for optimizing the CLSC network, balancing cost minimization with operational constraints to achieve an efficient and sustainable supply chain design.

## 4. Solution approach

The problem is classified as NP-hard, rendering exact methods impractical for finding a solution within a reasonable CPU time [41]. Therefore, this study employs the Artificial Bee Colony (ABC) algorithm, which has been extensively validated for its effectiveness, to solve this problem. This section provides a brief overview of the original ABC method, followed by a detailed explanation of the IHABC technique.

### 4.1. Basic description of ABC

The artificial bee colony algorithm (ABC), introduced by Karaboga and Basturk in 2005, is a swarm intelligence optimization technique designed to solve multidimensional and multimodal optimization problems by mimicking the foraging behavior of honey bees [31]. Since its inception, ABC has attracted significant attention from scholars [42,43]. The algorithm simulates the foraging behavior of bees, specifically focusing on three roles: employed bees, onlooker bees, and scout bees. A brief description is presented as follows:

#### 4.1.1. Population initialization.

In the initial phase, a population of *SN* solutions (food source positions) is randomly generated within the feasible solution space, where *SN* denotes the size of the population. The initial location of the food sources$x_{i}=\left(x_{i,1},x_{i,2},\cdots ,x_{i,D}\right),i=1,2,\cdots SN$ is generated as follows.


$$x_{i,j}=x_{min,j}+rand\cdot (x_{max,j}-x_{min,j})$$ (14)

Where i∈{1,2,⋯,SN},j∈{1,2,⋯,D}, *D* represents the dimension of the search space, and *rand* is a random value generated uniformly within the interval (0,1). $x_{max,j}$ and$x_{min,j}$ denote the upper and lower bound of each component, respectively. This initialization phase ensures that the algorithm starts with a diverse set of solutions, enabling a broad exploration of the search space. The subsequent phases of the algorithm, employed bees, onlooker bees, and scout bees, work collaboratively to refine these solutions, balancing exploration and exploitation to achieve near-optimal results.

#### 4.1.2. Employed bee phase.

In the employed bee phase, each bee searches around its assigned food source and generates a new food source $v_{i}=\left(v_{i1},v_{i2},\cdots ,v_{iD}\right)$ as below:


$$v_{i,j}=x_{i,j}+\phi_{ij}\cdot \left(x_{i,j}-x_{k,j}\right)$$ (15)

Wherei,k∈{1, 2, …, SN} and i≠k; $\phi_{ij}$ is a random number within [−1, 1]. If *v*_*i*_ outperforms the previous food source *x*_*i*_, *x*_*i*_ is replaced by *v*_*i*_ and the *counter* is reset to 0. Otherwise, *x*_*i*_ remains unchanged, and its *counter* is incremented by one. This process ensures that the algorithm continuously explores the search space while retaining high-quality solutions.

#### 4.1.3. Onlooker bee phase.

After the employed bee phase ends, the onlooker bees collect information shared by the employed bees. Each onlooker bee chooses a food source for further exploration, with the selection probability *p*_*i*_ determined as follows:


$$p_{i}=\frac{fit_{i}}{\sum_{i=1}^{SN}fit_{i}}$$ (16)

Where *p*_*i*_ represents the probability of selecting the employed bee and $fit_{i}$ is the fitness value of *i*th food source. Each onlooker bee moves to a food source *x*_*i*_, chosen using the roulette wheel selection strategy, and then uses Equation (15), to generate a new candidate food source *v*_*i*_. If *v*_*i*_ is better than *x*_*i*_, then *x*_*i*_ is replaced by *v*_*i*_, and its *counter* is reset to 0. Otherwise, *x*_*s*_ is retained, and its *counter* is incremented by one. This phase emphasizes exploitation by focusing on high-quality solutions while maintaining diversity through probabilistic selection.

#### 4.1.4. Scout bee phase.

In the scout bee phase, if the *counter* exceeds the predefined *limit*, the food source is abandoned, and the corresponding employed bee evolves into a scout bee, generating a new food source randomly according to Equation (14). After generating the new food source, its *counter* is set to 0, and the scout bee is converted into an employed bee. This phase ensures that the algorithm avoids stagnation by exploring new regions of the search space, thereby maintaining a balance between exploration and exploitation

### 4.2. IHABC for CLLIP

As noted in Section 4.1, ABC can be enhanced by adjusting its position update equation or by combining it with other intelligent optimization techniques. In this study, both methods are employed to improve population diversity and prevent the ABC algorithm from getting trapped in local optima, thereby enhancing its overall efficiency. The proposed algorithm, called IHABC, integrates ABC with the Differential Evolution Algorithm (DE). We have improved the standard ABC from two parts: (1) In the employed bee phase, a modified search equation (Equation 19), inspired by the DE/best/1 strategy, is introduced to enhance ABC’s exploitation capabilities and guide the search for candidate solutions. Additionally, a new control parameter, *τ*_1_, is introduced to balance exploration and exploitation. (2) In the onlooker bee phase, to enhance exploration and prevent premature convergence, the onlooker bee utilizes the DE/rand/1 strategy to exploit the current food position. Unlike Equation (19), a new search equation (Equation 20), incorporating the DE/rand/1 strategy and a selection probability *τ*_2_, replaces Equation (15).

The employed bee phase and the onlooker bee phase utilize a hybrid search strategy to address the limitations of the standard ABC algorithm. This hybrid approach ensures a more robust and efficient search process, combining the strengths of ABC and DE to achieve better solutions for the CLLIP. The definitions of the notations used are provided in Table 2.

**Table 2:** Notation in IHABC.

Notations	Explanation
*SN*	Population size (number of individuals)
*G*	The maximum generation for evolution
*K*	The number of successive iterations without improvement is reached
*S*	The number of suppliers
*I*	The number of customer zones
*R*	The number of HDCCs
*x*_*i*_^*g*^	The *i*th food source in generation *g* (1* ≤ i ≤ SN*; 1* ≤ g ≤ G*)
*v*_*i*_^*g*^	The *i*th new food source in generation *g* (1* ≤ i ≤ SN*; 1* ≤ g ≤ G*)
*τ*_1_	The modification rate for the employed bee phase
*τ*_2_	The selection probability for the onlooker bee phase

The key components of the IHABC for the CLLIP are discussed in the following subsections, covering population initialization, and the evolutionary search phases (employed bee, onlooker bee, and scout bee phases).

#### 4.2.1. Population initialization.

Designing an efficient encoding and decoding mechanism is crucial for enhancing algorithm performance, as it directly impacts the effectiveness and efficiency of the optimization process [44]. The encoding structure of each population individual plays a pivotal role in the algorithm’s operation, as it determines how solutions are represented and manipulated. By analyzing the model’s characteristics and uncovering its intrinsic properties, we design an encoding scheme tailored to the CLLIP. Each individual consists of vectors with *I* + *R* elements. The first *I* elements denote the candidate HDCC location strategy and the assignment information for the customer zones, while the last *R* elements indicate the candidate supplier location strategy and the allocation details for the HDCCs. The structure of the individual is given by Equation (17).


$$x_{i}^{g}=\left\{x_{i,1}^{g},x_{i,2}^{g},...,x_{i,I}^{g},x_{i,I+1}^{g},x_{i,I+2}^{g},...,x_{i,I+R}^{g}\right\},1\leq i\leq SN,1\leq g\leq G;$$ (17)

Where$x_{i}^{g}$ denotes the *p*-th solution at iteration *g*. $x_{i,j}^{g}\in \left\{1,2,\cdots ,R\right\}\left(1\leq i\leq SN;1\leq j\leq I\right)$ represents the identifier of HDCCs assigned to each customer zone, and $x_{i,j}^{g}\in \left\{1,2,\cdots ,S\right\}\left(1\leq i\leq SN;I+1\leq j\leq I+R\right)$ indicates the identifier of suppliers assi*g*ned to each HDCC. If $x_{i,j}^{g}=r\left(1\leq i\leq SN;1\leq j\leq I\right)$, then customer zone *j* is allocated to HDCC *r*, and if $x_{i,j}^{g}=s(1\leq i\leq SN;I\text{+}1\leq j\leq I+R)$, then candidate HDCC *j* is assigned to supplier *s*. Table 2 illustrates decoded individuals for a scenario involving ten customer zones, five candidate HDCCs, and three suppliers, providing a clear example of how the encoding and decoding mechanisms operate. This structured representation ensures that the algorithm can efficiently explore and evaluate solutions while maintaining feasibility and relevance to the CLLIP.

In Table 3, the decoded individual is structured to display the assignment information. The first part of the decoded individual shows the following assignments: customer zones 1, 4, and 8 are allocated to HDCC 1; customer zones 3, 5, and 9 to HDCC 3; customer zones 2, 6, 7, and 10 to HDCC 2; The second part reveals that candidate HDCCs 1, 2 and 4 are assigned to supplier 2, while candidate HDCC 3 and 5 are assigned to supplier 3. Thus, the decision variables *W*_*s*_, *X*_*r*_, *Y*_*sr*_, *Z*_*ri*_ can be determined based on the assignment information.

**Table 3:** Example of the decoded individual (*S*=3, *R* = 5, *I* = 10).

$x_{i,1}^{g}$	$x_{i,2}^{g}$	$x_{i,3}^{g}$	$x_{i,4}^{g}$	$x_{i,5}^{g}$	$x_{i,6}^{g}$	$x_{i,7}^{g}$	$x_{i,8}^{g}$	$x_{i,9}^{g}$	$x_{i,10}^{g}$	$x_{i,11}^{g}$	$x_{i,12}^{g}$	$x_{i,13}^{g}$	$x_{i,14}^{g}$	$x_{i,15}^{g}$
1	2	3	1	3	2	2	1	3	2	2	2	3	2	3

The purpose of encoding is to transform the initial population into a viable solution for the proposed model. By analyzing the encoding mechanisms of the population individuals and considering the specifics of the CLLIP, a specific decoding mechanism is derived, as shown in Equation (18).


$$\begin{array}{c} x_{i,j}^{g}=\left\{ \begin{array}{cc} \begin{array}{c} \;\mathrm{-30pt}round\left(x^{L}+rand\cdot \left(x^{U}-x^{L}\right)\right)\;1\leq j\leq I\\ round\left(y^{L}+rand\cdot \left(y^{U}-y^{L}\right)\right)\;I+1\leq j\leq I\text{+}R \end{array} & 1\leq i\leq SN,1\leq g\leq G; \end{array}\right. \end{array}$$ (18)

Where *round* ( ) denotes the rounding function, rand∈[0,1] represents a uniformly distributed random number. *x*^*L*^ and *x*^U^ denote the lower and upper bounds, respectively, with *x*^*L*^ = 1 and *x*^U^ indicating the total number of candidates HDCCs. Similarly, *y*^L^ and *y*^U^ denote the lower and upper bounds for the candidate suppliers, where *y*^L^ = 1 and *y*^U^ indicates the total number of candidate suppliers. This structured representation ensures that the algorithm can efficiently map the encoded solutions to the decision variables, enabling accurate evaluation and optimization of the CLLIP model.

#### 4.2.2. Employed bee phase.

To improve ABC’s performance, researchers have refined its search equations, resulting in the development of numerous variants. In IHABC, the DE/best/1 strategy is introduced to enhance exploitation and utilize information from the best solutions to guide candidate searches. A new candidate solution *v*_*i*_ is generated by using the best solution *x*_*best*_. A search equation, formulated based on the DE/best/1 strategy and the properties of ABC, is presented below:


$$\begin{array}{c} v_{i}^{,jg+1}=\left\{ \begin{array}{c} round(x_{b}^{est,jg}+\phi_{i,j}(x_{r}^{1,jg}-x_{r}^{2,jg})),rand<\tau_{1}\\ \;x_{b}^{est,jg},otherwise \end{array}.1\right.\leq g\leq G,1\leq i\leq SN,1\leq j\leq I+R; \end{array}$$ (19)

Where the function *round* () denotes the rounding operation. *x*_*best*_ is the best individual in the current population. *r*1 and *r*2 are integers randomly selected from {1, 2,..., SN}, and $\phi_{i,j}$ is a random number in the range [−1, 1]. *rand* is a uniformly distributed random number between [0, 1], while *τ*_1_ represents the modification rate, which takes a value between 0 and 1. If $v_{i}^{,jg+1}$ exceeds the pre-specified upper or lower bounds, it will be randomly regenerated within the respective range. This approach ensures that the search process is guided by high-quality solutions while maintaining diversity in the population.

#### 4.2.3. Onlooker bee phase.

The onlooker bee assesses the nectar information from all employed bees and selects a food source *x*_*i*_ based on its probability value *p*_*i*_. The probability of selecting a food source is defined by Equation (16) and utilizes the roulette wheel selection strategy, where solutions are chosen for the update process based on fitness proportionate selection. In IHABC, the update process in the onlooker bee phase differs from that in the employed bee phase. To improve the exploration capability of ABC and enhance the diversity of perturbed parameter vectors, a new search equation Equation (20), inspired by the DE/rand/1 strategy is presented to replace Equation (19). The selective probability *τ*_2_ is introduced to balance the exploration and exploitation of ABC. The new search mechanism is outlined below:


$$v_{i}^{,jg+1}=\left\{ \begin{array}{c} round\left(x_{r}^{1,ig}+\phi_{i,j}\left(x_{r}^{1,ig}-x_{r}^{2,ig}\right)\right),rand<\tau_{2}\\ x_{b}^{est,ig},otherwise \end{array}.1\right.\leq g\leq G,1\leq i\leq SN,1\leq j\leq I+R;$$ (20)

Where *round*() denotes the rounding operation, *r*1 and *r*2 are distinct random integers selected from {1, 2,..., *SN*}; *x*_*best*_ represents the best individual in the population; $\phi_{i,j}$ is a random number within the range of [−1, 1], indicating the change rate of the food source, which can affect the algorithm’s convergence speed.*τ*_2_ denotes the selective probability, ranging from 0 to 1. *rand* is a uniformly distributed random number within the interval [0, 1]. If $v_{i}^{,jg+1}$ exceeds the specified bounds, it will be randomly regenerated within the appropriate range. This hybrid search mechanism ensures a balance between exploration and exploitation, enabling the algorithm to escape local optima and converge to high-quality solutions.

#### 4.2.4. Scout bee phase.

In the scout bee phase, if a food source *x*_*i*_ cannot be improved after the predefined *limit*, it is abandoned, and the corresponding employed bee transitions to a scout. The scout then randomly generates a new food source as described in Equation (18).

Based on the previously described steps of population initialization, employed bee phase, onlooker bee phase, and scout bee phase, the pseudo-code for IHABC is presented below:

**Table:** 

Algorithm: Pseudo-code of IHABC
1:Set the parameters *SN*, *limit*, *G, iter = *0, *counter*(*i*) = 0 2:Generate the initial population $\left\{x_{i}|i=1,2\cdots ,SN\right\}$ using Equation (18) 3:Evaluate the function values of the population 4:**while** (*iter *< *G*) **do** 5: **for** (*i* = 1 to *SN*) **do** 6: Generate a new candidate solution *v*_*i*_ by Equation (19) 7: Calculate the objective function value *f*(*v*_*i*_) 8: **if** *f*(*v*_*i*_) < *f*(*x*_*i*_) **then** 9: *x*_*i *_= *v*_*i*_; *counter*(*i*) = 0 10: **else** 11: *counter*(*i*)= *counter*(*i*)+1 12: **end if** 13: **end for** 14:Calculate the probability value *p*_*i*_ by Equation (16), set *t *= 0, *i* = 1 15:** while** (*t* < *SN*) **do** 16: **if** *rand* <* p*_*i*_ **then** 17: Generate a new candidate solution *v*_*i*_ by Equation (20) 18: Calculate the objective function value *f*(*u*_*i*_) 19:** if** *f*(*v*_*i*_) < *f*(*x*_*i*_) **then** 20: *x*_*i *_= *v*_*i*_; counter(*i*) = 0 21: **else** 22: *counter*(*i*)= *counter*(*i*)+1 23:** end if** 24: *t* = *t* + 1 25: **end if** 26: *i* = *i *+ 1 27:** if** *i* = *SN* **then** 28:* i *= 1 29: **end while** 30: **If** *max*(*counter*(*i*))> *limit* **then** 31: Replace *x*_*i*_ with a new candidate solution by Equation (18) 32: ** End if** 33: Memorize the best solution achieved so far 34:* iter = it er+*1 35:**End while**

## 5. Numerical experiments

This section provides an experimental evaluation of the CLLIP model and the proposed algorithm, alongside a comparison of IHABC with other efficient algorithms. The evaluation starts with a detailed sensitivity analysis of IHABC’s parameters to determine their optimal settings. IHABC is then applied to solve the CLLIP model at different scales, and its performance is compared to that of ABC [31], GABC [43], CABC [42], qABC [45], and Lingo 11. Numerical experiments are conducted on three problem sizes: (1) small-scale (3 candidate suppliers, 5 candidate HDCCs, and 30 customer zones), (2) medium-scale (4 candidate suppliers, 15 candidate HDCCs, and 80 customer zones), and (3) large-scale (5 candidate suppliers, 20 candidate HDCCs, and 150 customer zones). Additionally, a sensitivity analysis is conducted to evaluate how key parameters (e.g., return rates, inventory costs, and transportation costs) affect the model’s performance. All experiments are implemented in JAVA on a Windows PC with an AMD Ryzen 5 3500U CPU, Radeon Vega Mobile Gfx GPU, 2.10 GHz, 16GB RAM, and Windows 11 OS. This comprehensive experimental setup ensures a rigorous evaluation of the proposed algorithm and model, providing valuable insights into their performance and scalability.

Since a standardized benchmark for CLLIP does not exist, several instances are generated using insights from previous studies. The MINLP model parameters are shown in Table 4, which are similar to that in Li et al. [25]. To represent the reality, *q*_*i*_ is set to 30% [4], and θ, *β* are set to 0.7, and 0.3, respectively, as observed by Guo et al. [29]. Candidate locations for suppliers, HDCCs, and customer zones are randomly placed within a 100 × 100 square, with shipping costs are proportional to the Euclidean distance between HDCCs and customer zones, reflecting real-world logistics scenarios where transportation costs are distance-dependent. This approach ensures that the generated instances are both realistic and representative of practical supply chain networks, enabling a meaningful evaluation of the proposed model and algorithm.

**Table 4:** Basic data for CLLIP.

Parameter	Value	Parameter	Value
*a*_*r*_	*U* [1000, 1500]	*a*_*s*_	*U* [1000, 1500]
*b*_*sr*_	*U* [5, 10]	*c*_*sr*_	*U* [5, 10]
*L*	1	*e*_*sr*_	5
*f*_*r*_	1	*h*_*r*_	*U* [1, 3]
*q*_*i*_	0.3	*µ*_*i*_	*U* [20, 30]
*σ*_*i*_^2^	*µ*_*i*_	*z*_*α*_	1.96
*β*	0.3	*λ*	300
*θ*	0.7		

U (a, b) is the uniform distribution over the interval of [a, b].

### 5.1. Parameters setting

Proper parameter setting is vital for achieving optimal performance of the algorithm. This section utilizes sensitivity analysis to evaluate the parameters of IHABC. In IHABC, the control parameters *τ*_1_ and *τ*_2_ significantly influence the search process and the quality of the solution. To determine the appropriate parameter values for *τ*_1_ and *τ*_2_, a parameter-tuning test is conducted. A test instance is created as a sample network with 4 candidate suppliers, 15 candidate HDCCs, and 80 customer zones. Each combination is executed 30 times, and the average total cost and CPU time (in seconds) are used as evaluation factors. Furthermore, to ensure fair comparisons and draw robust conclusions, the basic parameters of ABC-based algorithms are set to *SN* = 6*I*, *limit *= 5*I*, and *G *= 200. The numerical results are shown in Table 5.

**Table 5:** Parameters test τ_1_ and τ_2._

		τ_2_ = 0	τ_2_ = 0.1	τ_2_ = 0.2	τ_2_ = 0.3	τ_2_ = 0.4	τ_2_ = 0.5	τ_2_ = 0.6	τ_2_ = 0.7	τ_2_ = 0.8	τ_2_ = 0.9	τ_2_ = 1
τ_1_ = 0	mean	13529410.14	13556627.43	13575948.91	13560501.65	13632725.19	13663790.49	13731076.39	13783421.86	13887242.39	13974916.76	14048969.35
	CPU time	5.47	5.56	5.44	5.46	5.45	5.46	5.49	5.43	5.47	5.47	5.49
τ_1_ = 0.1	mean	13569156.14	**13517309.44**	13612055.01	13571813.86	13689797.07	13731119.21	13836551.27	13876242.57	14026651.98	14087307.99	14195239.62
	CPU time	5.45	5.49	5.44	5.59	5.43	5.49	5.46	5.44	5.51	5.48	5.48
τ_1_ = 0.2	mean	13596108.84	13604467.58	13631798.61	13667814.9	13714292.03	13804641.39	13891887.08	13925987.45	14115770.25	14227971.63	14375105.63
	CPU time	5.64	5.45	5.44	5.48	5.45	5.4	5.53	5.49	5.51	5.54	5.53
τ_1_ = 0.3	mean	13618389.14	13587544.77	13643249.86	13773899.68	13797274.56	13878071.8	14004249.52	14085126.71	14189287.7	14305824.74	14587681.68
	CPU time	5.48	5.48	5.45	5.49	5.44	5.54	5.54	5.48	5.59	5.55	5.51
τ_1_ = 0.4	mean	13627247.32	13665123.28	13701710.21	13816387.5	13868196.44	13979362.95	14095316.14	14192448.38	14379977.96	14545652.01	14675836.15
	CPU time	5.43	5.48	5.51	5.42	5.47	5.5	5.49	5.5	5.47	5.5	5.55
τ_1_ = 0.5	mean	13706100.82	13706083.3	13816167.85	13927556.14	14039686.91	14151108.49	14218896.02	14429757.6	14530642.35	14673989.23	15000783.83
	CPU time	5.47	5.52	5.51	5.47	5.47	5.41	5.48	5.49	5.49	5.45	5.47
τ_1_ = 0.6	mean	13688136.71	13822653.18	13838459.22	13985246.43	14132841.99	14250422.58	14365067.21	14634274.24	14729237.37	14942926.66	15253416.26
	CPU time	5.68	5.56	5.58	5.54	5.5	5.49	5.52	5.5	5.51	5.53	5.52
τ_1_ = 0.7	mean	13846939.22	13868855.81	14042768.12	14042635.54	14230829.85	14458826.49	14564243.44	14864977.02	15046327.38	15159492.25	15555233.43
	CPU time	5.66	5.47	5.47	5.53	5.58	5.49	5.53	5.46	5.43	5.51	5.51
τ_1_ = 0.8	mean	13954832.56	14098418.69	14124212.32	14147354.75	14394385.92	14597098.45	14893498.64	14952310.61	15266460.25	15560796.81	15858275.01
	CPU time	5.6	5.58	5.48	5.5	5.48	5.49	5.55	5.51	5.47	5.5	5.52
τ_1_ = 0.9	mean	13991071.51	14117976.25	14175764.12	14396602.06	14651196.38	14836111.75	15024919.9	15210084.45	15630293.79	15955450.07	16245516.08
	CPU time	5.7	5.48	5.53	5.47	5.53	5.43	5.55	5.49	5.49	5.5	5.6
τ_1_ = 1	mean	14150003.46	14219612.3	14384063.95	14656610.06	14821284.71	15101523.18	15217895.69	15515614.04	15798629.38	16165948.03	16633146.03
	CPU time	5.57	5.54	5.48	5.47	5.5	5.48	5.5	5.55	5.49	5.53	5.57

Based on the results presented in Table 4, the following observations can be made: (1) In terms of computational efficiency, variations in parameters *τ*_1_ and *τ*_2_ have a relatively minor effect on the algorithm’s runtime. (2) In terms of solution quality, variations in parameters *τ*_1_ and *τ*_2_ have a substantial impact on the quality of the solutions, significantly influencing the overall performance of IHABC.

Consequently, IHABC achieves optimal performance when *τ*_1 _= 0.1 and *τ*_2_ = 0.1 This setting will be used in the subsequent analysis to ensure the best possible results.

### 5.2. Efficiency of the proposed strategy

Since IHABC introduces new search strategies in both the employed bee and onlooker bee phases to improve ABC’s performance, it is important to rigorously evaluate the benefits of these new strategies to determine their contribution to algorithmic efficiency and robustness. In this section, the basic parameters of ABC-based algorithms are set to *SN* = 6*I*, *limit *= 5*I*, and *G* = 1000 for all instances, ensuring consistency across experiments. The search strategies are independently executed 30 times on small, medium, and large instances to ensure statistical reliability and generalizability of the results.

#### 5.2.1. Efficiency of the search strategy in the employed bee phase.

To validate the effectiveness of the proposed search strategy in the employed bee phase, the search equation from ABC is employed for comparison. To ensure a fair comparison, the two algorithms are identical except for the search strategy in the employed bee phase. The results of each instance are presented in Table 6.

**Table 6:** Comparison of improvement strategies results in the employed bee phase.

Instances	Search strategy	Min	Max	Mean	S.D.	CPU Time (Second)
Small scale	IHABC	8010546.38	8010546.38	8010546.38	0.00	1.72
	ABC	8010546.38	8010546.38	8010546.38	0.00	2.22
Medium scale	IHABC	12936564.50	12936564.50	12936564.50	0.00	24.40
	ABC	13808547.46	14525801.77	14216707.81	155564.39	27.15
Large scale	IHABC	21286027.06	21295385.72	21286974.56	1596.39	113.17
	ABC	28533491.99	29988263.77	29462014.18	371223.15	116.50

A summary of these results is provided below: (1) Compared with the search strategy of ABC, the proposed search strategy in the employed bee phase yields superior results, demonstrating enhanced optimization capabilities; (2) The CPU time values further indicate that the proposed search strategy achieves higher optimization efficiency, reducing computational resource requirements. (3) The lower standard deviation values associated with the proposed strategy indicate improved algorithmic stability and robustness, ensuring more consistent performance across iterations. (4) As the problem size increases, the advantages of the proposed strategy become increasingly pronounced, outperforming the original ABC strategy in larger and more complex instances.

Therefore, applying the proposed search strategy can significantly enhance the algorithm’s performance, as evidenced by its effectiveness in the employed bee phase.

#### 5.2.2. Efficiency of the search strategy in the onlooker bee phase.

To validate the performance of the proposed search strategy for the onlooker bee phase, the search equation derived from the ABC algorithm is employed as a benchmark for comparison. To ensure a fair assessment, the two algorithms are designed to be identical in all aspects except for the search strategy implemented during the onlooker bee phase. The results for each instance are presented in Table 7.

**Table 7:** Comparison of improvement strategies results in the onlooker bee phase.

Instances	Search strategy	Min	Max	Mean	S.D.	CPU Time (Second)
Small scale	IHABC	8010546.38	8010546.38	8010546.38	0.00	1.83
	ABC	8010546.38	8011654.55	8010628.09	0.00	2.22
Medium scale	IHABC	12936564.50	12936564.50	12936564.50	0.00	23.93
	ABC	13808547.46	14525801.77	14216707.81	155564.39	27.15
Large scale	IHABC	21285920.73	21291388.07	21286769.44	932.18	112.50
	ABC	28533491.99	29988263.77	29462014.18	371223.15	116.50

The results presented in Table 7 reveal that the proposed search strategy demonstrates significant advantages over the original ABC strategy across multiple dimensions. First, in terms of solution accuracy, the proposed search strategy significantly outperforms those of the original ABC approach, indicating its superior optimization performance and ability to identify more precise solutions. Second, while the improvement in solution efficiency is modest, the proposed search strategy still shows a slight improvement over the original, indicating that maintains competitive optimization speeds without compromising on quality. Third, the standard deviation values indicate that the proposed search strategy significantly enhances the algorithm’s stability, reducing variability in outcomes and ensuring more consistent and reliable performance. Finally, as the problem size increases, the advantages of the proposed search strategy become more pronounced, demonstrating its scalability and effectiveness in handling larger and more complex optimization tasks. These findings collectively underscore the effectiveness of the proposed search strategy for the onlooker bee phase, particularly in scenarios requiring high precision, stability, and scalability.

Managerially, the findings highlight the importance of integrating innovative methodologies into organizational decision-making frameworks. The proposed strategy’s ability to deliver consistent and reliable outcomes, particularly as problem complexity grows, makes it a valuable tool for industries such as logistics, manufacturing, and technology. By leveraging this approach, managers can improve operational efficiency, enhance strategic planning, and achieve more robust solutions to complex challenges. Moreover, the emphasis on stability and scalability underscores the need for organizations to invest in adaptive and forward-thinking strategies that can navigate the growing complexities of modern economic environments, ensuring long-term resilience and competitiveness.

### 5.3. Findings and discussion

#### 5.3.1. Performance comparison.

In this subsection, to further verify the problem-solving capabilities of IHABC, GABC, CABC, qABC, ABC, and Lingo are implemented. To ensure a fair and unbiased comparison, the basic parameters for the ABC-based algorithms are standardized as follows: *SN *= 6*I*, *limit* = 5*I*, and *G* = 1000 for all instances. For five instances of different scales, each algorithm is executed 30 times under identical conditions to ensure robustness and reliability in the results. Key performance metrics, including the minimum (min), maximum (max), mean, standard deviation (S.D.), and CPU time are recorded for comparison. As problem sizes increase, Lingo encounters significant computational challenges, failing to solve the proposed CLLIP model for medium and large-scale instances within a reasonable time limit of 3600 Seconds. Consequently, IHABC is compared with the other algorithms for medium and large-scale instances, with detailed results presented in Tables 8–10.

**Table 8:** Experimental results of different algorithms in small-scale instances.

Instance	Algorithm	Min	Max	Mean	S.D.	Time (Second)
Small scale-1	IHABC	8010546.38	8010546.38	8010546.38	0.00	1.68
	CABC	8010546.38	8010546.38	8010546.38	0.00	1.70
	qABC	8010546.38	8010546.38	8010546.38	0.00	1.79
	GABC	8010546.38	8010546.38	8010546.38	0.00	2.12
	ABC	8010546.38	8010546.38	8010546.38	0.00	2.22
	Lingo	8010546.00				1742.00
Small scale-2	IHABC	8233453.77	8233453.77	8233453.77	0.00	1.67
	CABC	8233453.77	8233453.77	8233453.77	0.00	1.70
	qABC	8233453.77	8233453.77	8233453.77	0.00	1.80
	GABC	8233453.77	8233453.77	8233453.77	0.00	2.18
	ABC	8233453.77	8234263.32	8233533.28	197.47	2.39
	Lingo	8233636.00				1592.00
Small scale-3	IHABC	8538707.94	8538707.94	8538707.94	0.00	1.65
	CABC	8538707.94	8538707.94	8538707.94	0.00	1.68
	qABC	8538707.94	8538707.94	8538707.94	0.00	1.78
	GABC	8538707.94	8538707.94	8538707.94	0.00	2.13
	ABC	8538707.94	8538707.94	8538707.94	0.00	2.24
	Lingo	8538708.00				2209.00
Small scale-4	IHABC	9694848.83	9694848.83	9694848.83	0.00	1.67
	CABC	9694848.83	9694848.83	9694848.83	0.00	1.67
	qABC	9694848.83	9694848.83	9694848.83	0.00	1.83
	GABC	9694848.83	9694848.83	9694848.83	0.00	2.09
	ABC	9694848.83	9694848.83	9694848.83	0.00	2.19
	Lingo	9694849.00				1694.00
Small scale-5	IHABC	8293509.72	8293509.72	8293509.72	0.00	1.68
	CABC	8293509.72	8293509.72	8293509.72	0.00	1.70
	qABC	8293509.72	8293509.72	8293509.72	0.00	1.78
	GABC	8293509.72	8293509.72	8293509.72	0.00	2.11
	ABC	8293509.72	8294905.41	8293670.90	416.63	2.29
	Lingo	8293510.00				1691.00

**Table 9:** Experimental results of different algorithms in medium-scale instances.

Instance	Algorithm	Min	Max	Mean	S.D.	Time (Second)
Medium scale-1	IHABC	12936564.50	12936564.50	12936564.50	0.00	22.09
	CABC	12936564.50	12936564.50	12936564.50	0.00	23.33
	qABC	12936564.50	12936564.50	12936564.50	0.00	24.13
	GABC	13203301.31	13754085.34	13585288.89	128911.51	27.00
	ABC	13808547.46	14525801.77	14216707.81	155564.39	27.15
Medium scale-2	IHABC	12716008.34	12716688.14	12716234.94	320.46	22.71
	CABC	12716008.34	12716688.14	12716302.92	336.87	24.21
	qABC	12716008.34	12716688.14	12716438.88	327.59	23.64
	GABC	13342365.94	13947427.21	13763251.93	122769.71	27.07
	ABC	13967516.64	14926772.53	14436299.03	201685.92	27.20
Medium scale-3	IHABC	13944312.19	13944312.19	13944312.19	0.00	21.75
	CABC	13944312.19	13944312.19	13944312.19	0.00	23.43
	qABC	13944312.19	13944312.19	13944312.19	0.00	23.83
	GABC	14336441.31	14677279.60	14479948.31	85891.20	26.90
	ABC	14945907.03	15477232.87	15212619.58	146878.32	27.35
Medium scale-4	IHABC	13709651.02	13709760.43	13709702.08	54.58	22.26
	CABC	13709651.02	13709760.43	13709659.92	27.52	23.77
	qABC	13709651.02	13709760.43	13709669.62	35.00	23.95
	GABC	14404515.25	14759910.68	14601841.28	74550.14	27.26
	ABC	14680487.05	15301321.57	15036460.27	150114.46	27.07
Medium scale-5	IHABC	14202477.54	14202804.38	14202645.06	122.11	22.11
	CABC	14202477.54	14202742.55	14202651.05	103.03	23.43
	qABC	14202477.54	14202742.55	14202654.83	118.25	23.26
	GABC	14789828.89	15108187.94	14963188.76	89534.72	27.18
	ABC	14829759.11	15849840.82	15516424.76	197837.48	27.26

**Table 10:** Experimental results of different algorithms in large-scale instances.

Instance	Algorithm	Min	Max	Mean	S.D.	Time (Second)
Large scale-1	IHABC	21285601.07	21286027.81	21285917.18	64.44	105.46
	CABC	21285676.86	21286742.34	21285996.63	171.42	112.05
	qABC	21678707.53	22350720.56	22095640.72	135069.21	116.44
	GABC	27386837.10	29296071.53	28513220.22	347123.62	117.18
	ABC	28533491.99	29988263.77	29462014.18	371223.15	116.50
Large scale-2	IHABC	23295511.44	23295702.14	23295553.74	53.64	106.70
	CABC	23295511.44	23296331.88	23295676.35	202.74	112.12
	qABC	23804108.76	24431485.79	24076688.28	151029.94	116.49
	GABC	30135311.10	30963833.52	30534340.13	192712.76	117.79
	ABC	30489556.84	31971588.13	31279338.21	345818.77	116.77
Large scale-3	IHABC	22732196.90	22732572.39	22732472.48	58.71	104.58
	CABC	22732303.04	22732713.87	22732573.80	113.94	111.50
	qABC	23319093.15	24007463.33	23608596.04	176894.73	116.95
	GABC	30101331.27	31991342.01	31434231.45	469755.34	117.00
	ABC	31435033.50	33130366.05	32461802.51	440322.52	117.26
Large scale-4	IHABC	25051114.80	25051480.92	25051468.71	65.72	104.16
	CABC	25050770.25	25051480.92	25051457.23	127.57	112.57
	qABC	25382483.99	25773171.75	25603053.42	92750.92	117.47
	GABC	30750119.39	32255636.91	31531648.46	352175.85	116.95
	ABC	31329729.46	33410659.08	32662063.22	402884.67	116.93
Large scale-5	IHABC	24144361.47	24144919.51	24144820.43	161.90	103.16
	CABC	24144565.59	24144895.44	24144867.92	81.79	111.42
	qABC	24704713.17	25355245.74	24904014.05	132949.17	117.18
	GABC	31854126.82	33196401.99	32544716.99	356526.51	117.24
	ABC	31613504.45	34619614.45	33923828.85	550590.00	117.68

The results for the performance comparison are presented in Tables 8–10, which reveal the following key conclusions:

(1) For small-scale instances, IHABC and Lingo produce nearly identical optimal solutions, highlighting IHABC’s robust search capability and its ability to match the performance of established optimization tools. Moreover, IHABC consistently outperforms Lingo in terms of computational efficiency across all instances, highlighting its superior ability to deliver results in less time.
(2) IHABC consistently exhibits superior solution quality compared to other algorithms, as indicated by its lowest minimum and mean values across all instances. This highlights IHABC’s remarkable capability, effectiveness, and robustness in addressing the proposed CLLIP, making it a reliable choice for complex optimization problems.
(3) IHABC demonstrates notable consistency, with the lowest standard deviations in solution values among all algorithms. This reliability ensures steady performance, making IHABC particularly suitable for applications that require stable and dependable results, such as real-time decision-making or mission-critical operations.
(4) IHABC demonstrates outstanding computational efficiency, requiring less CPU time than other algorithms. This efficiency makes it particularly advantageous in practical scenarios where both computational resources and time are limited, enabling faster and more cost-effective solutions.

A *t*-test was applied to assess whether the performance difference of IHABC is statistically significant. Due to the minor impact on small-scale instances, the test was conducted on medium and large-scale instances. A *t*-test with a 95% confidence interval was used to determine if there is a significant difference in total cost and to provide statistical evidence of the IHABC’s performance. The test results for medium-scale and large-scale instances are presented in Tables 11 and 12.

**Table 11:** The results of *t*-test for medium-size problems.

	Medium size-1		Medium size-2		Medium size-3		Medium size-4		Medium size-5	
	*t*-value	*p-*value	*t*-value	*p-*value	*t*-value	*p-*value	*t*-value	*p-*value	*t*-value	*p-*value
IHABC	–	–	-0.787	0.434	–	–	3.714	0.001	-0.202	0.841
CABC										
IHABC	–	–	-2.397	0.020	–	–	2.696	0.010	-0.310	0.758
qABC										
IHABC	27.100	0.000	45.926	0.000	33.583	0.000	64.444	0.000	45.744	0.000
GABC										
IHABC	44.315	0.000	45.927	0.000	46.501	0.000	55.103	0.000	29.912	0.000
ABC										

**Table 12:** The results of *t*-test for large-size problems.

	Large size-1		Large size-2		Large size-3		Large size-4		Large size-5	
	*t*-value	*p-*value	*t*-value	*p-*value	*t*-value	*p-*value	*t*-value	*p-*value	*t*-value	*p-*value
IHABC	-2.302	0.029	-3.205	0.003	-4.095	0.000	0.425	0.674	-1.360	0.184
CABC										
IHABC	32.287	0.000	-27.853	0.000	-26.672	0.000	-32.020	0.000	-30.751	0.000
qABC										
IHABC	112.121	0.000	202.267	0.000	-99.753	0.000	-99.089	0.000	126.867	0.000
GABC										
IHABC	118.607	0.000	124.315	0.000	118.994	0.000	101.727	0.000	-95.639	0.000
ABC										

Tables 11 and 12 show that the *p*-value for most problems is less than 0.05. This indicates that, in most cases, the IHABC algorithm significantly outperforms the other algorithms, with a notably lower total cost. Therefore, IHABC is a more efficient method, providing better feasible solutions.

#### 5.3.2. Sensitivity analysis.

**Fig 2:**
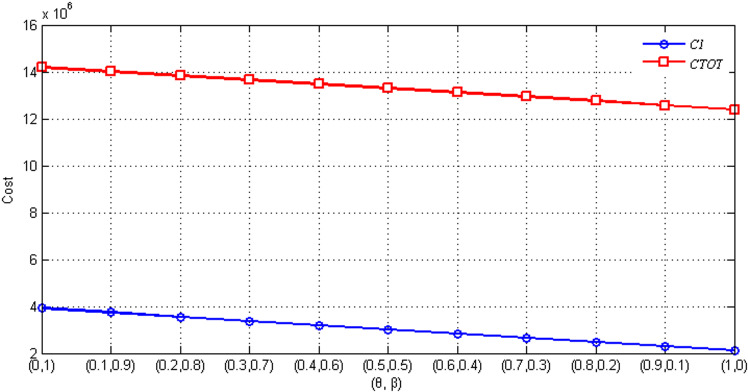
Sensitivity analysis on (*θ*, *β*).

**Fig 3:**
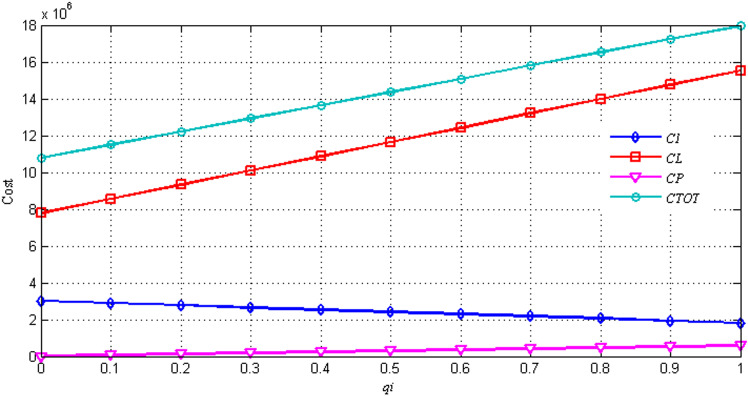
Sensitivity analysis on *q*_*i*_.

In this section, we analyze the impact of three critical parameters - return rate *q*_*i*_, false failure return rate *θ*, and true failure return rate *β* - on the costs in the proposed model, including location and delivery cost (*C*_*L*_), inventory cost (*C*_*I*_), and processing cost (*C*_*P*_). The results are shown in Figs 2 and 3, which provide valuable insights into the relationships between these parameters and the associated costs.

In Fig 2, *q*_*i*_ is held constant while *β* and *θ* are varied. Since the parameters affecting distribution and recycling costs are predetermined, and both *C*_*L*_ and *C*_*P*_ are independent of *β* and *θ*, the analysis focuses on how changes in *β* and *θ* influence *C*_*I*_ and total cost (*C*_*TOT*_). As *θ* increases and *β* decreases, both *C*_*I*_ and *C*_*TOT*_ decline. This trend can be attributed to the higher rate of false failure returns allowing more products to be reintegrated into the market via HDCCs, thereby reducing ordering and inventory costs. As a result, the system achieves better resource utilization and significant cost savings, highlighting the economic benefits of optimizing return management processes.

In Fig 3, with (*θ*, *β*) fixed at (0.7, 0.3), *q*_*i*_ varies within the range [0, 1]. The results indicate that as *q*_*i*_ increases, *C*_*L*_, *C*_*P*_, and *C*_*TOT*_ rise, while *C*_*I*_ decreases. This trend is likely driven by the fixed demand: as the number of returned items increases, both return handling costs and distribution costs between the HDCC and the demand point escalate. Conversely, the ordering and inventory costs between the supplier and the HDCC decrease, leading to a reduction in *C*_*I*_ as *q*_*i*_ increases. This inverse relationship underscores the trade-offs inherent in managing return rates and their impact on overall system costs. Additionally, the findings highlight the need for flexible supply chain strategies that can adapt to varying return rates, ensuring that organizations remain competitive in dynamic market environments.

In the preceding section, the impact of return rates, as well as the rates of true and false failure returns, on associated costs were analyzed, yielding several management insights. First, given that inventory costs decline as return rates increase, companies should adopt more flexible inventory management strategies, such as dynamic inventory adjustments, to alleviate inventory backlogs and their associated costs. This approach not only enhances operational efficiency but also aligns inventory levels more closely with actual demand, reducing waste and improving resource utilization. Second, companies can achieve significant cost savings by improving product quality and reducing the frequency of true failure returns. Achieving this may require strategic investments in research and development (R&D) and enhancements in production processes, which can lead to long-term competitive advantages and higher customer satisfaction. Third, the time and costs associated with processing returns can be minimized by optimizing the returns process. For instance, automating the returns processing system can streamline operations, improve efficiency, and reduce labor costs, ultimately enhancing the overall profitability of the return management system. Fourth, the rate of false failure returns can be reduced by improving customer education and technical support. This can be achieved through measures such as providing comprehensive product usage manuals, offering troubleshooting assistance, and ensuring easily accessible customer service hotlines, all of which contribute to a better customer experience and fewer unnecessary returns. Finally, managers can leverage historical return data to analyze and forecast future return trends. This data-driven approach enables proactive adjustments in inventory and production plans, thereby helping to reduce excess inventory and associated costs while ensuring that supply chain operations remain agile and responsive to market demands.

Furthermore, from an economic perspective, these insights highlight the importance of optimizing return management processes to achieve cost efficiency and operational excellence. By reducing true failure returns and minimizing false failure returns, companies can lower their overall costs while improving customer satisfaction and brand reputation. Furthermore, investments in R&D and process automation not only enhance product quality and operational efficiency but also contribute to long-term sustainability and competitiveness in the market. Ultimately, these strategies enable companies to transform return management from a cost center into a value-adding component of their operations, driving both financial and operational success.

#### 5.3.3. Discussion.

This research introduces the improved Hybrid Artificial Bee Colony (IHABC) algorithm to tackle the complex multi-supplier closed-loop location-inventory problem (CLLIP) with customer returns. The algorithm aims to minimize the total supply chain costs, considering key factors such as location and delivery costs, inventory costs, and processing costs. The numerical experiments and sensitivity analyses conducted in this study offer valuable insights into the operational implications of various parameters and their impact on overall supply chain performance. The key findings are as follows:

(a) *Complex supply chain management and cost reduction:* The supply chain environment, especially in industries with high return rates, presents significant challenges related to facility location and inventory control. The complexity is further compounded by the need to handle a considerable volume of returned products, which directly affects operational efficiency. The IHABC algorithm proves to be an effective tool for addressing these challenges, providing viable solutions that optimize facility locations and inventory management strategies. By reducing both location and inventory costs, the algorithm allows businesses to manage returns more effectively and achieve cost savings without sacrificing service levels. The results suggest that IHABC offers a practical and robust solution to managing closed-loop supply chains, particularly in industries where returns are a significant concern.
(b) *The role of recycling in enhancing profitability:* Customer returns are an inherent part of many supply chains, especially in sectors like e-commerce. These returns, however, can be both costly and complex, involving multiple products and supply chain nodes. The study emphasizes the importance of enhancing the recycling process to mitigate the impact of returns. By improving recycling practices, companies can reintegrate returned products into the supply chain, thereby boosting sales and profitability. Recycling not only contributes to reducing waste but also ensures that companies can maintain competitive advantages in a dynamic and highly competitive market. The findings highlight that managers must focus on optimizing recycling strategies to manage returns effectively, improve product life cycles, and maximize the profitability of returned items.
(c) *Sensitivity of costs to return rate* (*q*_*i*_): The sensitivity analysis reveals that the return rate (*q*_*i*_) has a significant effect on supply chain costs. As the return rate increases, distribution and handling costs escalate, thereby increasing location and processing costs (*C*_*L*_ and *C*_*P*_). However, at the same time, inventory costs (*C*_*I*_) tend to decrease due to lower demand, as returns replace the need for new orders. This inverse relationship underscores the complex nature of managing returns within a supply chain and suggests that companies must carefully balance the return rate to avoid unnecessary cost increases. Therefore, firms should implement flexible supply chain strategies that can dynamically adjust to varying return rates. These strategies could include adaptive inventory management systems, dynamic demand forecasting models, and cost-sensitive return handling protocols that optimize return-related expenditures.
(d) *Optimization of return management:* The study’s sensitivity analysis further provides crucial insights into optimizing returns management processes. By minimizing both true and false failure returns, companies can significantly reduce processing costs and improve their overall cost structure. The analysis indicates that a well-designed return management strategy can lead to substantial cost savings, as it minimizes the need for redundant processes and enhances operational efficiency. For example, automating the returns process can speed up the handling of returned items, reducing labor costs and improving the accuracy of return processing. Additionally, a streamlined return process enables companies to reintegrate returned products back into the inventory more quickly, reducing inventory holding costs and improving product availability.
(e) *IHABC’s superior performance:* The numerical comparisons between IHABC and other solution methods demonstrate the algorithm’s superior performance in solving the multi-supplier CLLIP. IHABC not only provides better feasible solutions in terms of total cost (*C*_*TOT*_) but also does so with fewer computational resources. This makes the algorithm particularly valuable in scenarios where computational efficiency is crucial, such as real-time decision-making processes in dynamic supply chain environments. The results clearly show that IHABC can help recycling companies and other businesses dealing with complex location-inventory problems achieve cost reductions while maintaining high service levels. Furthermore, the flexibility of the IHABC allows it to be applied to other combinatorial optimization problems, particularly those involving supply chain optimization, where both location and inventory management play crucial roles.
(f) *Strategic implications for supply chain management:* From a broader perspective, the findings of this research suggest several strategic implications for managers in industries affected by high return rates. First, companies should focus on improving product quality to reduce true failure returns, which can be costly in terms of both processing and inventory costs. Investments in R&D and process improvements can contribute to product durability and reduce the frequency of returns, thereby lowering overall costs. Second, given the substantial impact of return rates on cost structures, companies need to adopt more agile and responsive inventory management systems that can quickly adjust to changes in demand and return patterns. This includes the use of predictive analytics to forecast future return trends, allowing firms to plan better for potential surpluses or shortages of returned products.

Finally, the economic benefits of optimizing return management go beyond mere cost reduction. By improving the returns process, companies can increase customer satisfaction by offering quicker and more seamless return experiences. This, in turn, can enhance brand loyalty and increase the likelihood of repeat business. Furthermore, by transforming return management from a cost center into a value-adding operation, companies can improve both their profitability and operational sustainability.

## 6. Conclusions and future research

This study proposes a mixed-integer nonlinear programming model to address the multi-supplier closed-loop location-inventory problem (CLLIP) in the context of customer returns. To solve this complex problem, an improved Hybrid Artificial Bee Colony (IHABC) algorithm is developed by integrating novel search strategies in the employed bee and onlooker bee phases to effectively balance exploration and exploitation within the ABC framework. Furthermore, numerical experiments validate the superior performance of IHABC compared to the ABC-based algorithms and Lingo, demonstrating its ability to identify optimal solutions more efficiently across small, medium, and large-scale instances. Notably, IHABC excels in handling large-scale instances, showcasing its robustness and scalability in addressing real-world optimization challenges. Additionally, a sensitivity analysis of critical model parameters, such as returns and failure rates, provides actionable management insights for practitioners, enhancing the practical applicability of the findings in real-world scenarios.

The findings of this study provide several valuable managerial contributions, particularly for industries grappling with high return volumes, such as e-commerce, retail, and manufacturing. By optimizing the integration of location and inventory decisions in a closed-loop supply chain, the proposed IHABC-based model helps organizations achieve significant cost reductions, improve resource utilization, and enhance operational resilience. From a practical standpoint, the model’s ability to balance the trade-off between location and inventory costs in the context of customer returns is crucial for enhancing supply chain efficiency. Furthermore, the insights derived from the sensitivity analysis, particularly the effects of return rates and failure rates on costs, offer actionable recommendations for managers. Understanding how changes in these parameters influence overall costs enables organizations to develop more flexible and adaptive supply chain strategies that can quickly adjust to dynamic market conditions. For example, by managing return rates effectively, firms can reduce processing and distribution costs, while simultaneously improving customer satisfaction through a more efficient returns management process.

Additionally, the study underscores the importance of managing returns as a critical component of a broader supply chain strategy. Companies should adopt an integrated approach that considers not only the economic factors but also operational factors like product quality, logistics, and customer service. Implementing automated systems for returns processing, leveraging predictive analytics for demand forecasting, and incorporating feedback loops into the return management system can substantially improve profitability and customer retention. In this context, the findings are especially relevant for businesses seeking to stay competitive in industries where customer returns are a significant concern, particularly during peak seasons or economic downturns.

This study has certain limitations, such as not addressing supply chain uncertainties like demand fluctuations and disruption risks, and focusing solely on economic factors. Future research can explore: (1) the impact of deep uncertainties, including market demand fluctuations and return quantity variations; (2) disruption risks from supplier or transportation interruptions and their effects on the supply chain; and (3) the integration of social and environmental factors, such as carbon emissions, waste reduction, and corporate social responsibility, to enhance sustainability and real-world applicability.

## Abbreviations

CLSC: Closed-loop supply chain
HDCC: Hybrid distribution-collection center
ABC: Artificial B bee colony
IHABC: Improved hybrid artificial bee colony
CABC: Artificial bee colony with candidate solutions
qABC: quick Artificial bee colony
GABC: Global-best-guided artificial bee colony
DE: Differential evolution algorithm
GA: Genetic algorithm
LIP: Location-inventory problem
CLLIP: Closed-loop location-inventory problem
MINLP: Mixed-integer nonlinear programming problem
